# Towards Inference of a Biochemical Ontology From a Metabolic Database

**DOI:** 10.1002/cfg.500

**Published:** 2005

**Authors:** D. C. McShan

**Affiliations:** School of Medicine University of Colorado Health Sciences Center PO Box 6511 Mail stop 8303 Aurora CO 80045-0511 USA

## Abstract

In order to predict the metabolic fate of an arbitrary compound based solely on structure, it is useful to be able to identify substructural ‘functional groups’ that are biochemically reactive. These functional groups are the substructural elements that can be removed and replaced to transform one compound into another. This problem of identifying functional groups is related to the problem of classifying compounds. The research presented here discusses the state of the art in biochemical databases and how these sources may be applied to the problem of classifying compounds based solely on structure. We describe a biochemical informatics system for processing molecular data and describe how 100 255 compositional (hasA) relationships are inferred between 835 abstractions and 9500 metabolites from the KEGG Ligand database. Specifically, we focus on the identification of amino acids and consider ways in which the inference of biochemical ontologies for metabolites will be improved in the future.


**Comparative and Functional Genomics**
*Comp Funct Genom* 2005; **6**: 398–406.
Published online in Wiley InterScience (www.interscience.wiley.com). **DOI:** 10.1002/cfg.500

**Research Article**

### Towards inference of a biochemical ontology from a metabolic database

D. C. McShan*
*School of Medicine, University of Colorado Health Sciences Center, Aurora, CO, USA*

*Correspondence to:
D. C. McShan, School of Medicine, University of Colorado Health Sciences Center, PO Box 6511, Mail stop 8303, Aurora, CO 80045-0511, USA.
E-mail: Daniel.McShan@UCHSC.edu*

Received: 28 September 2005
Revised: 1 December 2005
Accepted: 5 December 2005

### Abstract

**In order to predict the metabolic fate of an arbitrary compound based solely on structure, it is useful to be able to identify substructural 'functional groups' that are biochemically reactive. These functional groups are the substructural elements that can be removed and replaced to transform one compound into another. This problem of identifying functional groups is related to the problem of classifying compounds. The research presented here discusses the state of the art in biochemical databases and how these sources may be applied to the problem of classifying compounds based solely on structure. We describe a biochemical informatics system for processing molecular data and describe how 100 255 compositional (hasA) relationships are inferred between 835 abstractions and 9500 metabolites from the KEGG Ligand database. Specifically, we focus on the identification of amino acids and consider ways in which the inference of biochemical ontologies for metabolites will be improved in the future. Copyright © 2006 John Wiley & Sons, Ltd.**

**Keywords:** **Ontology; classification; molecular structure; substructure search**

### Introduction

An ontology is a hierarchical classification system that is used to organize information. Ontologies are especially useful when combined with annotated data for making inferences about the data. For metabolites, we are generally interested in how they may be transformed, both chemically and enzymatically. Biochemists look at molecules and identify abstractions called functional groups, which are substructural components that are chemically reactive in some fashion. There are two intrinsic aspects that define this reactivity. The first is the functional group itself, and the second is the rest of the molecule. Both are solely a function of the molecule's structure.

We hypothesize that biochemical reactivity of arbitrary compounds can be inferred directly from their structure and, furthermore, that the information to accomplish this already exists in metabolic databases such as KEGG Ligand [10] and MetaCyc [11], but remains untapped. The current research endeavours to generate a method for computing a compositional ontology for compounds in these databases.

### Metabolic resources

Metabolic databases such as KEGG Ligand and SRI's BioCyc ostensibly facilitate the storage and delivery of molecular data. In practice, though, while these ontologies may exist, there has been almost no effort to apply them as molecular classifiers. There is no way, for instance, to automatically classify a new compound in the system. As an example, consider one of the simplest classifications, that the compound ethanol is an alcohol. Even simpler still, that it has an alcohol. This relationship between ethanol and alcohol does not formally exist in any of the common molecular ontologies.

GO [7], for instance, has an entry for neither ethanol nor for the abstraction alcohol. A quick search on 'alcohol' in GO identifies 10 processes, 34 functions, and no components. This may not come as a surprise, since this is an ontology of genes, after all, and not small molecules.

Copyright © 2006 John Wiley & Sons, Ltd.

KEGG, the 'encyclopedia' of genes and genomes, does have a metabolic component, Ligand. Ligand has several entries for the class ALCOHOL, including PRIMARY ALCOHOL, SECONDARY ALCOHOL, ARYL ALCOHOL, etc. Ligand also has instances for ethanol and other alcohols, but there is no class–instance relationship. Indeed, they are all equivalent object types — ultimately nothing more than database entries.

Over the past several years, SRI has developed a series of knowledge bases (Eco-Cyc, Bio-Cyc, Meta-Cyc) and has evolved an ontology which can be used for reasoning over them. These ontologies are 'inheritance' ontologies, and encode 'isA' type classifications. In fact, the AN ALCOHOL concept does exist as a class. It has two instances, *cis*-3-hexenol and *trans*-2-hexenol. In MetaCyc, the AN ALCOHOL class isA UNCLASSIFIED COMPOUND, and MetaCyc does have an instance for ethanol, but it is also classified separately under UNCLASSIFIED COMPOUNDS and has no relation to 'AN ALCOHOL'.

It is worth noting that in the MeSH [1] classification, ethanol is a child of ALCOHOLS. In MeSH, however, the parent–child relationship is not obviously an 'isA'-type relationship. Consider that ETHANOLAMINES is a child of ETHANOL, and the statement 'epinephrine is a ethanol' is not entirely true. Clearly there is some relationship, but it is more of a substructural 'hasA'-type relationship. MeSH, of course, has no chemical structure and no links to other data sources. However, NCBI has a database of compounds called PubChem [2], which does have links to the MeSH tree for specific compounds such as ethanol, but not for classes such as alcohol.

The closest attempt to embody this knowledge is probably the Klotho system, developed by Kazic [12]. Klotho can describe molecules explicitly by their substituents, but it only has 439 compounds and ethanol is not one of them. Fortunately, the manual uses it as an example. Klotho uses configuration rules to describe molecules. Thus, for ethanol, the config rule is:

config ('ethanol',
[chain ([hydroxymethyl, methyl])]).

Hydroxymethyl is like alcohol, but it is not really encoded in a fashion suitable for our purposes. Moreover, this data source seems to have been manually curated, and has no automated method for generating the config rules directly from structure.

Recently, EBI has recently adopted Ashburner's chemical ontology as ChEBI [6]. The ontology here is also a directed acyclic graph and we can see, for instance, that ETHANOL is a great-grandchild of ALCOHOLS. It is interesting to note that ETHANOL is a child of ETHANOLS, and is sibling to CHLOROETHANOLS and (1S)-1-PHENYLETHANOL. PHENYLETHANOL is a child of ETHANOL in MeSH. ChEBI does contain links to KEGG for specific instances such as ETHANOL, and recently has added abstract classes such as ALCOHOL and ETHANOL. Since ChEBI is expert-curated, it should provide an excellent truth model for evaluating our classification methods in the future.

Additionally, there are several chemical informatics methods which identify functional groups within arbitrary molecules for the purpose of assessing reactivity. Indeed, this is one of the primary concepts in retrosynthetic analysis [8], and is used extensively in computational chemistry. Quite recently, Feldman *et al.* [9] have discussed utilizing a substructural search approach to automatically infer a chemical ontology. This approach uses the program 'checkmol' [3] to infer function group compositions in a target molecule.

### Abstract compounds

The concept AN ALCOHOL is an example of what we refer to as an abstract compound. Both KEGG and Eco-Cyc databases have abstract compounds, but they are implemented slightly differently. Whereas KEGG provides them on equivalent semantic status with concrete compounds, MetaCyc uses them as classifications. The advantage of this is the MetaCyc has an ontology based on these classifications, but the disadvantage is that it is manually populated (or not, as the case may be). Importantly, the KEGG approach has curated Markush (R-group) structural representations for these abstract compounds, whereas MetaCyc has only a few structures. The amino acid abstraction, for instance, has no structure in MetaCyc. Thus, it is curation which we intend to exploit for our automated inference of a biochemical ontology.

### Project goals

The goal of this current research is to automate compositional classifications for these types of databases for these abstractions. As a first step, this

Copyright © 2006 John Wiley & Sons, Ltd. 

project will explore the computation of substructural relationships, under the hypothesis that structure determines composition. Specifically, we are in search of is Substructure Of and has Substructure relationships between compounds in KEGG. While substructural composition does not conclusively imply functional composition, we hypothesize that it is necessary but probably not sufficient. Regarding sufficiency, consider that GLUCOSE has five HYDROXYL groups, and while GLUCOSE may be an ALCOHOL chemically, it may not react as such in a biochemical milieu. As a particular goal of this project, we will consider the classification of amino acids and see whether we can identify their instances using substructural search. A quick search in KEGG indicates abstract compounds for $\alpha$-AMINO ACID, AMINO ACID, L-AMINO ACID, D-AMINO ACID and AROMATIC AMINO ACID. While we will compute over the entire corpus, our specific aim is to properly classify the standard amino acids.

### Methods

The initial step is to parse the KEGG data into our knowledge base. The version of Ligand that we are using dates from 12 January 2004. For this research, we are only focusing on the file of compounds (compound) and the directory of structures (mol/). The compound file contains 10 668 compounds (in contrast, the current version contains 11 092 entries); however, only 9660 have structures. The file was parsed using the BioPython Martel parser, which needed to be updated to reflect the current fields.

This data was then parsed into the Protege [4] knowledge base (KB), using the Jython [5] scripting language interfaced to the Protege API. Jython provides mechanisms for overloading nearly every aspect of the language grammar, including the attribute accessors. The molecular structures are provided by KEGG Ligand as mol files. They are read with a Jython script that uses the Chemaxon JChem library. Originally this data was converted to Simplified Molecular Input Line Entry Specification (SMILES) and stored as a slot in the KB. However, due to a bug in the Protege–Postgres backend, strings longer than 256 characters are not stored. As a result of this, and to increase computational efficiency, the JChem Molecule Java objects are stored in a Jython dictionary (hash table).

There are two compositional (hasA) relationships that we are interested in deriving: first, the is Substructure Of slot between a compound and those compounds that contain it; second, in the knowledge base an inverse has Substructure slot is defined. The inverse slot is maintained by the Protege software. All that is needed for population is the is Substructure Of slot instances. The search algorithm is straightforward — we search every compound against every other. If a match occurs, we add an instance of the is Substructure Of slot for the query frame.

Since there are 10 688 compounds in KEGG, it stands to reason that there are $10\,688^2$ substructure searches to perform to comprehensively identify all the relationships. This is roughly 100 million searches, and was considered to be intractable considering the time allocated for this research. By focusing on the abstract compounds as queries, it is hoped that the search space will be drastically reduced. Previous analysis of the compounds in Ligand indicated that there are approximately 800 such abstractions, reducing the number of searches to around eight million. Furthermore, previous unpublished observations indicate that many of the smaller abstractions (e.g. ALCOHOL) had isomers in the database, and an uninformed search would result not only in repeated substructure searches but in a significant consumption of time. To improve efficiency, a flag is stored in the KB, indicating that its search is complete. As the search progresses through the targets, if an isomer is found and it has a completed search, then its results are copied to the current query and the algorithm proceeds to the next query.

### Results

There are 9660 molecular structures in the KEGG release we used. Of these, there are 835 abstract substructures. We exhaustively search these patterns against every molecule in the database. Without optimizations, the theoretical number of searches would be $835 \times 9660 = 8\,066\,100$ searches. However, with the optimizations that consider isomers, in practice it amounts to 8 064 766. The searches (with database access) take about 11 h single-threaded on a dual 2.0 Ghz G5. From these searches, we identified 120 455 substructural relationships. For the hasSubstructure relationship,

Copyright © 2006 John Wiley & Sons, Ltd. 

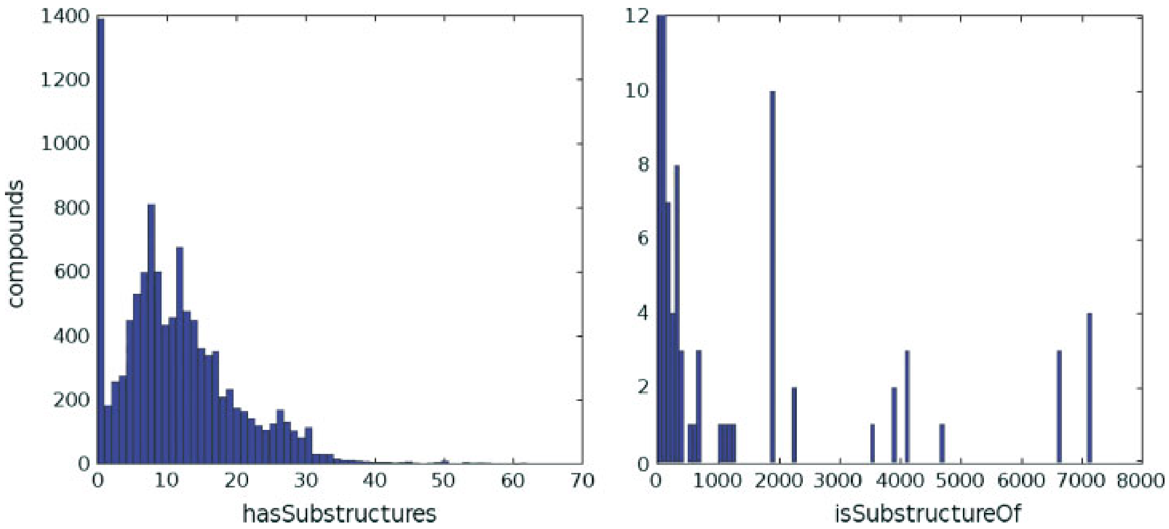

**Figure 1.** Distributions of slot values for re and is Substructure Of slot. In both cases, the predominant frequency is 0. For has Substructures, 1168 compounds have 0 substructures, 9500 have at least one (and on average about 11) substructures. The is Substructure Of slot was only computed for the 835 abstract compounds, and as a result, the distribution is significantly sparser. If these 835, 433 are substructures of at least one compound. Given the distribution, averages are not very meaningful

9500 compounds have at least one substructure; there are an average of 11.29 compounds containing each substructure, with a standard deviation of 8.22, median of 10, min of 0 and a max of 61 (tRNA). For the is Substructure relationship, we only computed values for the 835 abstract compounds. Of these, 433 were substructures of at least one compound. Four substructures (alcohol and three isomers) had 7090 containing compounds. The distributions for both slots are shown in Figure 1.

### The amino acid compositional ontology

We specifically wanted to consider the composition of AMINO ACIDS. Figure 2 illustrates a comparison of the MetaCyc ontology for AMINO ACIDS (left) with that inferred by substructure (right). MetaCyc has the protein-building amino acids curated separately from the so-called AMINO ACID DERIVATIVES, even though many of the derivatives are, in fact, amino acids. For instance, D-AMINO ACIDS are classified as derivatives.

The inferred tree on the right has slightly different semantics, and this is indicated by the change in arrow notation. The MetaCyc relations are inheritance-type relationships, and we use the UML notation for this type of relationship (an arrow at the parent class). Thus an AMINO ACID is A SMALL MOLECULE. Furthermore, MetaCyc instances (enumerated in the boxes to the right) are instances of that class. Thus GLYCINE isA AMINO ACID.

The semantics for the graph on the right are that of isA, and we use the UML notation for composition (diamond at the composing object). This compositionality relationship is quite different from the inheritance relationship in MetaCyc. Effectively, while ALPHA-AMINO ACID isA COMPOUND, we say that L-AMINO ACID isA ALPHA-AMINO ACID, and further that GLYCINE isA ALPHA-AMINO ACID. The distinction (and notation) may seem subtle but the gulf between composition and inheritance is quite a bit larger than we had previously thought, as discussed below.

Table 1 shows the hasSubstructure relationships for the common, protein-making amino acids. These are also the instances classified by MetaCyc as amino acids. The goal was to ensure that these known instances at least have the necessary (but not sufficient) substructures. In fact, all the amino acids do contain the ALPHA-AMINO ACID pattern except for PROLINE, which is problematic, as we discuss below.

In terms of the chiral patterns, there is no classification potential based on this data: all the L-amino acids also have the D-pattern. Furthermore,

Copyright © 2006 John Wiley & Sons, Ltd. 

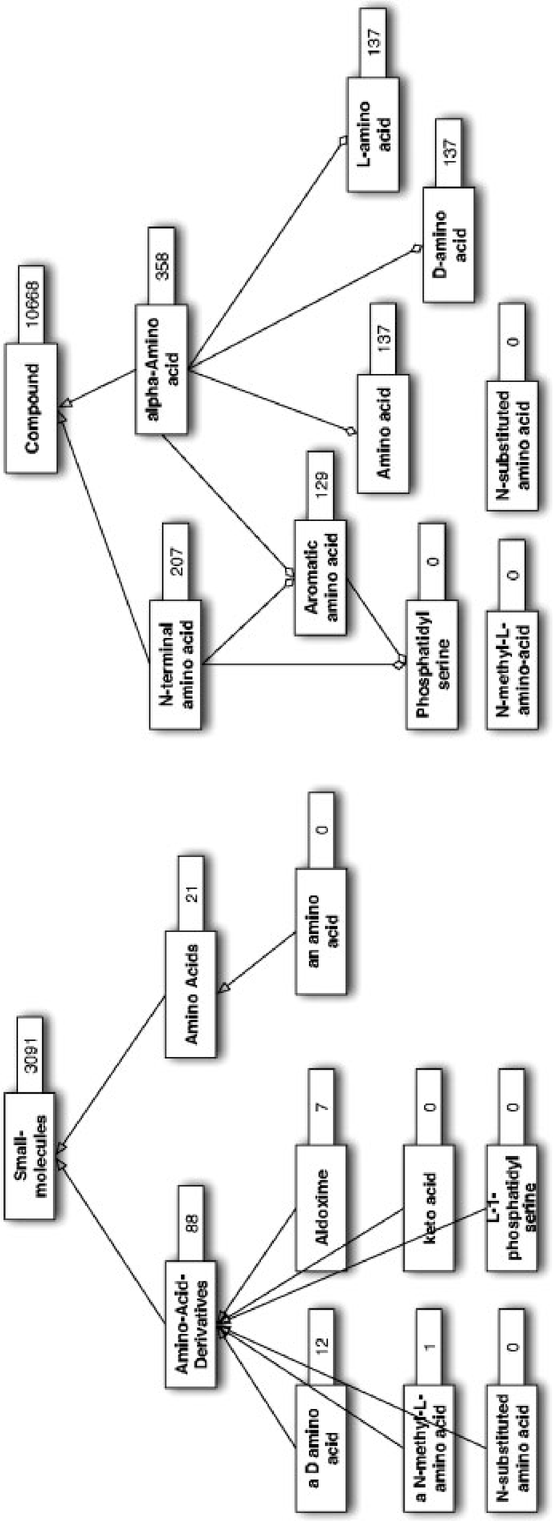

**Figure 2.** Comparison of the MetaCyc ontology with the substructural relationships discovered in this paper

**Table 1.** Identification of standard amino acids by substructural relationships

| | | A | R | N | D | C | E | Q | G | H | I | L | K | M | F | P | S | T | W | Y | V |
|---|---|---|---|---|---|---|---|---|---|---|---|---|---|---|---|---|---|---|---|---|---|
| | **KEGG ID** | – | – | – | – | – | – | – | – | o | – | – | – | – | o | – | – | – | o | o | – |
| $\alpha$ | C05167 | × | × | × | × | × | × | × | × | × | × | × | × | × | × | | × | × | × | × | × |
| AA | C00045 | | | × | × | | | | | × | | × | | | | | | | | | |
| D | C05405 | | | × | × | | | | | × | | × | | | | | | | | | |
| L | C00151 | | | × | × | | | | | × | | × | | | | | | | | | |
| Aro | C01021 | × | × | | | × | × | × | × | | × | | × | × | × | | × | × | × | × | × |

Aromatic R-groups are indicated by o, aliphatic by — . $\alpha$ is the alpha-amino acid frame, AA is the amino acid frame, D- and L- are the chiral frames, and Aro- is the aromatic amino acid frame. Substructural composition is sufficient to classify these compounds as amino acids. Only proline is not identified as an amino acid by substructural content, which is not surprising, since proline is not technically an amino acid — due to the >NH structure it is an imino acid. Nevertheless, in the text is some discussion about opportunities for discovering this functional classification. The substructure search algorithm does not distinguish chirality, although the abstractions do have chiral information, neither does it correctly predict aromaticity.

the aromatic predictions, while they do seem to classify all the aromatic amino acids, are plagued by false positives (e.g. ALANINE is obviously not AROMATIC).

### Discussion

While these results are certainly mixed, overall this project was a success. Eight million searches were performed in a reasonable amount of time, and some very useful relationships were identified. Furthermore, the environment for storing and manipulating this data was very effective and offers great potential for future biochemical investigations.

Obviously, all 120 455 relationships were not validated; after a preliminary review, most of them appear to be reasonable. Selecting the specific goal of investigating amino acids was fortuitous, and the results are quite interesting and informative. It would seem that our hypothesis was quite naive, and that substructural composition is not even necessary for functional classification. The reasons for this are discussed below.

### Composition is insufficient

The prime example for false positives is the alcohol substructure discussed above. We identify 7090 compounds which putatively have the alcohol substructure. In fact, there are four isomers of alcohol in the KEGG database: C00069, ALCOHOL; C01335, ROH; C02525, ALIPHATIC ALCOHOL; and C03130, LOWER PRIMARY ALCOHOL. All have the structure R-OH, i.e. hydroxyl.

Of course, not all hydroxyl groups are alcohols. Water, for instance, is obviously not an alcohol despite the fact that it has the OH substructure. Clearly, a knowledge of the 'R' part — the environment — is necessary to establish whether the substructure is chemically active. This is not terribly surprising, and really just confirms our hypothesis that composition is not *sufficient* to determine function.

### Composition is not even necessary

While substructural composition is clearly not 'sufficient', we hypothesized that substructural composition might at least be 'necessary' — specifically, that all amino acids would at least have the amino acid substructure. In fact, this is true for all of the amino acids — except proline. The fact that proline does not have an ALPHA-AMINO ACID is obviously problematic for our assertion that substructural composition is necessary for functional classification. Clearly PROLINE is an AMINO ACID — but, in fact, it is not. It is actually an IMINO ACID because of its >NH group (see Figure 3). There is no IMINO ACID compound abstraction in any of the databases. PROLINE is identified as having an AROMATIC ACID (although it is not actually aromatic — more on this later) as well as a SECONDARY (and TERTIARY) AMINE, as the amino acid pattern requires, but the structures do not match because of the extra N bond.

The problem here is that proline is *functionally* an amino acid but is not *structurally* one. The distinction is more than just semantic. This raises an important consideration, viz. what is 'function' and what is a 'functional group'? Specifically,

Copyright © 2006 John Wiley & Sons, Ltd. 

what is an amino acid? From a chemist's point of view, the amino acid is precisely defined as simply an acid with an amino group, and in fact, in our search, proline has both the acid and the amino. Biochemically, however, amino acids are the building blocks of proteins, and this is not a clearly structural definition.

### What is necessary?

It is interesting to consider what substructures are at least candidates for being necessary, that is, which substructures exist in every amino acid. Surprisingly, as shown in Table 2, there are 13, most of which from a biochemical perspective are

| *Name* | *SMILES* |
|---|---|
| α-Amino acid | NC(-[ $ (H,*])])C(O)=O |
| Amino acid | N[C@@H](-[ $ ([H,*])])C(O)=O |
| D-Amino acid | N[C@H](-[ $ ([H,*])])C(O)=O |
| L-Amino acid | [H][C@@](N)(-[ $ ([H,*])])C(O)=O |
| Aromatic amino acid | N[C@@H](-[ $ ([H,*])])C(O)=O |
| Glycine | NCC(O)=O |
| L-Alanine | C[C@H](N)C(O)=O |
| L-Proline | CC.CC.OC(=O)[C@H]1CCCN1 |
| L-Histidine | NC(Cc1c[nH]cn1)C(O)=O |

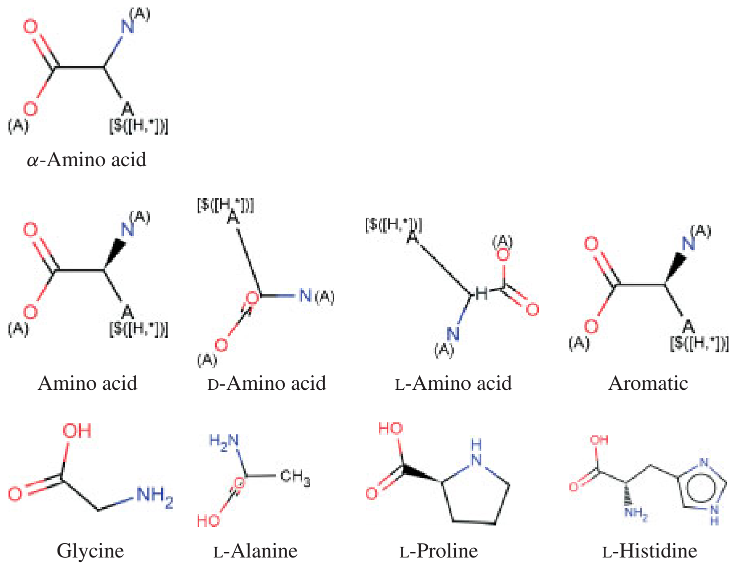

**Figure 3.** SMILES and structural representations for representative amino acids discussed in the text. The structures for D- and L- amino acid are rendered with a 3D projection to demonstrate chirality. The =O in D-amino acid is indicated as coming out of the page, whereas the =O in L-amino acid is coplanar with the −O and the N. The rest are laid out in 2D. In fact, the 'amino acid' frame is clearly identical to L-amino acid. There is no difference in the SMILES representations between the amino acid and the presumable aromatic variety. Yet, the algorithm does distinguish some amino acids as aromatic and some as not

**Table 2.** Substructures that are present in all 20 common amino acids

| | | | |
|---|---|---|---|
| Alcohol | Fatty acid | Ester | Aromatic acid |
| [O;A][R] | OC([R])=O | [R]OC([R])=O | [O;A]C([R])=O |
| Ketone | ROH | R−CO−R | Dialkyl ketone |
| C([R])=O | ROHO[R] | [R]C([R])=O | [R]C([R])=O |
| Tertiary amine | Secondary amine | Carboxylic ester | Aliphatic alcohol |
| N([R])[R] | [R]N[R] | [R]OC([R])=O | O[R] |
| Lower primary alcohol | | | |
| O[R] | | | |

Copyright © 2006 John Wiley & Sons, Ltd. 

clearly not necessary. While CARBOXYLIC ACID is not common, the CARBOXYLIC ACID ESTER substructure is present, and it is comforting to note that all the amino acids at least have SECONDARY (and TERTIARY) AMINES.

### Chirality

Despite these results, in theory we still believe that we should be able to infer chirality using the substructures. As shown in Figure 3, the SMILES representation would seem to distinguish between these abstractions. The D- and L-amino acid pattern explicitly expresses the stereochemistry about the primary carbon. In the SMILES nomenclature, this is represented by an ampersand. A single ampersand (@) indicates that the other three atoms are listed anti-clockwise, two ampersands indicate clockwise (@@). This is discussed further below with respect to the source mol files. In practice, the search engine does not seem to acknowledge the chiral specification. As a result, compounds that have the L-form also have the D-form.

### Aromatic classification

The aromatic substructural classification is clearly wrong. Unlike with chirality, where it merely failed to distinguish between two classes, the aromatic classification is incorrect. All the aromatic amino acids do have the aromatic substructure. As shown in Figure 3, there really is nothing in the aromatic substructure to identify an aromatic R-group. In fact, we now believe that aromatic amino acids cannot be identified simply by substructural composition, since the aromatic class is really a classifier of the R-group.

### R-group descriptors

There are formally two parts to a Markush R-group specification. There is the specification of the specific part (the pattern), and then there also needs to be specification of the R-portion. The original usage has the phrase 'where R is a'. This concept needs to be reintroduced into the world of enzyme nomenclature and classification. Clearly, alcohol dehydrogenase enzymes do not work on all hydroxyl groups. Effectively, what is required here is a pharmacophore model for the enzymes. The model might be structural, but it might also be rule-based. One can easily imagine determining reactivity based upon electrochemical parameters. This would better define what is meant by these abstractions. A simple pharmacophore model could define, for instance, that the R-group of aromatic amino acids should, in fact, be aromatic. This problem is likely prevalent in more than just the substructures we investigated.

### Summary

There is a need for biomolecular ontologies and, in particular, ones that have sufficient knowledge to classify metabolites automatically. With this in mind, we explored the substructural relationships between abstract and concrete compounds in KEGG. We developed an elegant system for computing over molecular data, primarily using the Jython programming language and the Protege knowledge base. Using this system, we identified over 120 000 relationships between substructural abstractions and other compounds. Specifically, we examined the amino acid relationships. Our hypothesis that substructural composition would be necessary for functional classification appears to be in error. The problem is highlighted by the fact that we would like to classify proline as an amino acid, in spite of the chemical evidence, which suggests that it is not. We also discovered and discussed problems in matching stereochemistry and specifying aromaticity. The stereochemistry issue will likely be resolved by the search engine, whereas the aromaticity issue points to a more difficult problem, one which will require classification of the R-group as well as the substructures. Despite these problems, we consider this project to be a success and the infrastructure developed should be sufficient (if not necessary) to solve these and other interesting problems in curating and applying metabolic knowledge.

### Acknowledgements

This research was performed with Dr Imran Shah. The work was conducted using the Protege resource, which is supported by Grant LM007885 from the United States National Library of Medicine. The engineers at ChemAxon made this research possible through their generous time and support of the JChem library.

Copyright © 2006 John Wiley & Sons, Ltd. 

### References

1. URL **http://www.nlm.nih.gov/mesh/meshhome.html**.
2. URL **http://pubchem.ncbi.nlm.nih.gov/**.
3. URL **http://www.sciencedirect.com/science?_ob=Redirect-URL&_method=externObjLink&_locator=url&_cdi=4938&_plusSign=%2B&_targetURL=http%253A%252F%252Fhttp://merian.pch.univie.ac.at/nhaider/cheminf/cmmm.htmlhttp://merian.pch.univie.ac.at/nhaider/chem-inf/cmmm.html**.
4. URL **http://protege.stanford.edu/**.
5. URL **http://www.jython.org/**.
6. Brooksbank C, Cameron G, Thornton J. 2005. The European Bioinformatics Institute's data resources: towards systems biology. *Nucleic Acids Res* **33**: (database issue): D46–53. doi: 10.1093/nar/gki026. URL **http://dx.doi.org/10.1093/nar/gki026**.
7. The Gene Ontology Consortium. 2000. Gene ontology: tool for the unification of biology. *Nature Genet* **25**: 24–29.
8. Corey EJ, Long AK, Rubenstein SD. 1985. Computer-assisted analysis in organic synthesis. *Science* **228**(4698): 408–418.
9. Feldman HJ, Dumontier M, Ling S, Haider N, Hogue CWV. 2005. CO: a chemical ontology for identification of functional groups and semantic comparison of small molecules. *FEBS Lett* **579**(21): 4685–4691. doi: 10.1016/j.febslet.2005.07.039. URL **http://dx.doi.org/10.1016/jfebslet.2005.07.039**.
10. Goto S, Nishioka T, Kanehisa M. 1998. LIGAND: chemical database for enzyme reactions. *Bioinformatics* **14**(7): 591–599.
11. Krieger CJ, Zhang P, Mueller LA *et al.* 2004. MetaCyc: a multiorganism database of metabolic pathways and enzymes. *Nucleic Acids Res* **32**: (database issue): D438–442. doi: 10.1093/nar/gkh100. URL **http://dx.doi.org/10.1093/nar/gkh100**.
12. Holcomb Wise JWB, Kazic T. 2000. *Klotho: A Tutorial and Manual*. IBC technical report. URL **http://www.biocheminfo.org/klotho/manual/klotho.html**.

Copyright © 2006 John Wiley & Sons, Ltd. 
